# The influence of a specific ophthalmological electronic health record on ICD-10 coding

**DOI:** 10.1186/s12911-016-0340-1

**Published:** 2016-07-26

**Authors:** Karsten Kortüm, Christoph Hirneiß, Michael Müller, Alexander Babenko, Anselm Kampik, Thomas C. Kreutzer

**Affiliations:** University Eye Hospital, Ludwig-Maximilians-University, Mathildenstrasse, 8, D-80336 Munich, Germany

**Keywords:** EHR, Electronic health record, EMR, Electric medical records, ICD-10, Ophthalmology, Introduction, ICD-10 coding

## Abstract

**Background:**

A specific Electronic Health Record (EHR) for ophthalmology was introduced in an academic center in Germany. As diagnoses coding corresponding to the International Classification of Diseases Version 10 (ICD-10) is mandatory for billing reasons in Germany, we analyzed whether a change occurred in the diversity and number of diagnoses after the EHR introduction. The number of patients was also analyzed. Proper diagnoses coding is of the utmost importance for further data analysis or billing.

**Methods:**

Graphical User Interfaces (GUIs) were created by using Advanced Business Application Programming language in EHR “i.s.h.med.” Development of an EHR was conducted in close collaboration between physicians and software engineers. ICD-10 coding was implemented by using a “hit list” and a search engine for diagnoses. An observational analysis of a 6-month period prior to and after the introduction of an ophthalmological specific EHR was conducted by investigating the diversity and number of diagnoses in various ophthalmological disease categories and the number of patient consultations.

**Results:**

During the introduction of a specific ophthalmological EHR, we observed a significant increase in the emergency department cases (323.9 vs. 359.9 cases per week), possibly related to documentation requirements. The number of scheduled outpatients didn’t change significantly (355.12 vs. 360.24 cases per week). The variety of diagnoses also changed: on average, 156.2 different diagnoses were made per week throughout our hospital before the EHR launch, compared to 186.8 different diagnoses per week thereafter (*p* < 0.05). Additionally, a significantly higher number of diagnoses per case and per week were observed in both emergency and subspecialty outpatient clinics (1.15 vs. 1.22 and 1.10 vs. 1.47, respectively).

**Conclusions:**

An optimized EHR was created for ophthalmological needs and for simplified ICD-10 coding. The implementation of digital patient recording increased the diversity of the diagnoses used per case as well as the number of diagnoses coded per case. A general limitation to date is the suboptimal precision of ICD-10 coding in ophthalmology. Correct coding is of utmost importance for future data analysis.

**Electronic supplementary material:**

The online version of this article (doi:10.1186/s12911-016-0340-1) contains supplementary material, which is available to authorized users.

## Background

Healthcare providers increasingly implement Electronic Health Records (EHR) in their hospitals [1]. Evidence is available that EHR helps deliver better healthcare and to increase cost-effectiveness [2]. In most situations, a Hospital Information Systems (HIS) is linked to an Enterprise Resource Planning software (ERP). For administrative reasons and cost reduction, many institutions focus on one comprehensive EHR system and refuse to use specialized subsystems. Typically, most software solutions only allow the sufficient recording of general medical and surgical data that are not specific for respective specialties. Capturing special data effectively in small specialties such as ophthalmology is not supported usually. This is especially unfortunate, as ophthalmology is a subspecialty with a large number of outpatients generating a huge amount of numeric data (e.g., visual acuity, and intraocular pressure). A recent study has identified ophthalmology together with dermatology and psychiatry as being the medical specialties with the lowest use of EHRs [3].

Recently, governments have started to offer incentives for the introduction of EHRs. To date, many countries worldwide use the International Classification of Diseases (ICD) version 10 coding. The employment of an EHR is of utmost importance for the proper digital coding of diseases. German hospitals have been obliged to code every admitted case by using ICD-10-based diagnoses since the year 2000. A German modification (ICD-10-GM) has been used since 2004 and differs from the original suggestion of the World Health Organization (WHO) in the suffixes. The ICD-10-clinical modification (CM) proposed by the American Center for Disease Control and Prevention (CDC), which has been used in the USA only since October 2015, is also slightly different from the first WHO proposal [4].

In ophthalmology, the specific ICD-10 codes use the prefix H00 to H59, stratified by anatomy. Traumas carry a “T” prefix, and injuries an “S” prefix. The legend to Table 1 gives examples of the ICD-10 categorization.

**Table 1:** Number of diagnoses per anatomical ICD-10 category

Anatomical ICD-10 category	Period 1	Period 2	Absolute change	Change (%)
H00 - H06	2,028	2,528	500	24.65
H10 - H13	1,816	2,275	459	25.28
H15 - H22	2,393	3,072	679	28.37
H25 - H28	1,068	1,617	549	51.40
H30 - H36	3,163	4,814	1,651	52.20
H40 - H42	837	1,084	247	29.51
H43 - H45	662	936	274	41.39
H46 - H48	289	390	101	34.95
H49 - H52	764	992	228	29.84
H53 - H54	224	314	90	40.18
H55 - H59	93	113	20	21.51
B30	380	493	113	29.74
T15.0	477	486	9	1.89
S05.0	623	366	−257	−41.25
S05.1	355	307	−48	−13.52
Z01.0	1,649	1,161	−488	−29.59
Z46.0	5	16	11	220.00
Z96.0	301	1,132	831	276.08
E14	1,240	1,199	−41	−4.4

Legend Table 1: H00-H06: Affection of eyelid, tear organ, and orbit; H10-H13: Affection of the conjunctiva; H15-H22: Affection of the sclera, cornea, iris, and ciliary body; H25-H28: Affection of the lens; H30-H36: Affection of uvea and retina; H40-H42: Glaucoma; H43-H45: Affection of the vitreous and globe; H46-H48: Affection of the optical nerve; H49-H52: Affection of eye muscles, strabismus, refractive errors; H53-H54: Visual impairment and blindness; H55-H59: Other affections of the eye and adnexa; B30.0: Viral conjunctival infection; T15.0: Corneal foreign body; S05.0: Injury of cornea without foreign body; S05.1: Contusion of the globe; Z01.0: Visual acuity and eye examination; Z46: Prescription of glasses and contact lenses; Z96.1: Presence of intraocular lens implant; E14: Ophthalmological diabetic complications

In 2004, HIS (i.s.h.med, Cerner GmbH) was implemented at the University Hospital of the Ludwig-Maximilian University (LMU) in Munich for administrative purposes. The Department of Ophthalmology of the LMU is a major regional academic ophthalmic healthcare provider with over 65,000 outpatient and 12,000 inpatient cases annually. As no specific ophthalmological user interfaces were available for the used HIS, a team of ophthalmologists and computer scientists started to adapt this system for effective use in the department in 2012. The expectation was to increase efficiency by instant access to medical documentation and to avoid time-consuming searches for missing files. As the number of patients continues to increase, an optimized workflow supported by the corresponding digital processing of their data is needed. Furthermore, special attention was paid to developing a medical-user- and clinical-workflow-orientated software, concurrently capturing as much administrative data as possible (e.g., ICD-10). Proper coding quality is not only relevant for reimbursement, but also offers a great opportunity for retrospective research in a short time, as digital data is easily and quickly available. Patients also benefit from good coding quality as they can be asked to participate in clinical trials, and other treating physicians can maintain an overview of correct diagnoses.

The aim of this study is to evaluate the change by numbers and diversity in recorded ICD-10 diagnoses because of the implementation of a specific ophthalmological EHR system. Moreover, the change in the number of patients has been analyzed pre- and post-EHR introduction.

## Methods

### Digital data input

The EHR system used in our project was i.s.h.med ERP 6.0 (Cerner GmbH, Erlangen, Germany), which is based on the software solution “Industry Solution – Healthcare” (IS-H) (SAP AG, Walldorf, Germany). I.s.h.med is an HIS system with over 300 installations worldwide, predominantly in larger healthcare providers in Europe and the Middle East. As the software was originally designed for administrative purposes in the 1990s, users often report limited usability. Programming is carried out in an advanced business application programming language (ABAP), which is exclusive to SAP, but resembles Common Business Oriented Language (COBOL) first introduced in 1959 [5]. It is possible to create defined graphical user interfaces (GUI), which are named parametric medical documents (PMDs).

Full-time development of GUIs started in January 2013 [6]. The first step was to create a general outpatient PMD. This was adapted to the clinical workflow in order to support clinicians in their daily work and to reduce time for documentation by eliminating the need to retrieve paper charts and to re-evaluate a patient’s past medical history in cases in which a paper chart goes missing. In addition, for each treatment, a semi-automatic report can be generated.

As part of the EHR implementation process, we did perform training for the use of the EHR in general. There was no special emphasis placed on the coding process and its benefits. All the staff members involved (residents, consultants, nurses and administrative staff) were trained.

The PMD was designed to make full use of the hospital’s standard 22-inch screen without the need for scrolling during data input or reading by using tabs.

The first three tabs capture the clinically most important data, structured by the examination workflow. The last three tabs contain surgical reports, other reports such as ultrasound imaging, and the possibility of creating a letter without the need for repetitive entries. In the first tab, the past medical history and current complaints of the patient are entered (Additional file 1: Figure S1). The second tab (Additional file 2: Figure S2) contains fields for visual acuity values to be entered either as a decimal or in letters, as defined in the early treatment of diabetic retinopathy study (ETDRS) [7]. Data entries are validated; e.g., only certain values can be entered for visual acuity.

**Fig. 1:**
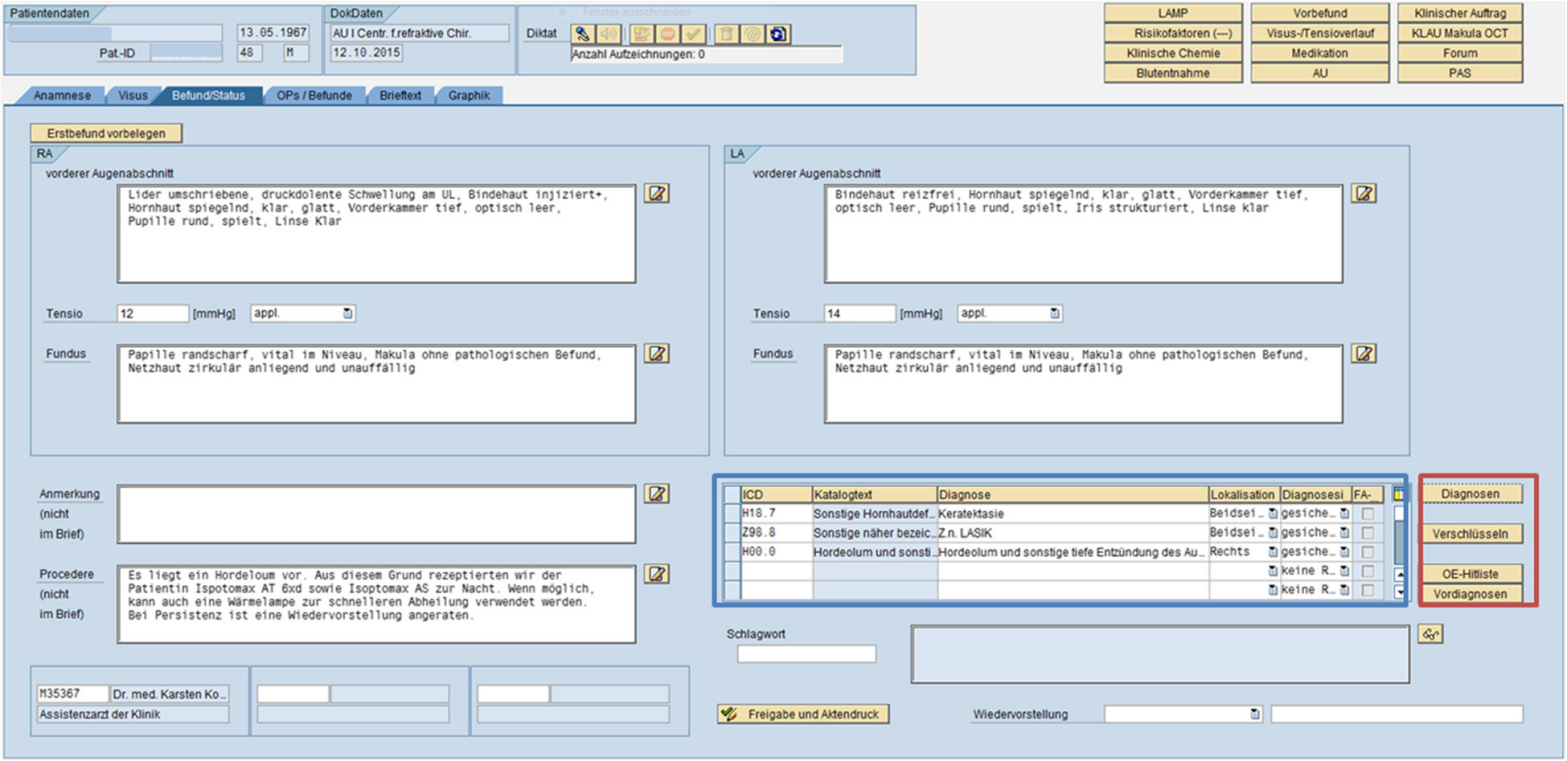
This is the third and most important tab of the EHR and allows recording clinical findings and treatment plans. In the lower right a table for entering diagnoses (*blue box*) is placed. Four buttons are located to the right of the table (*red box*). The first button allows entering ICD-10 diagnoses directly, if the code is known, the second button opens a diagnoses search engine, the third button opens our hit list in a pop-up window and the last button allows transferring diagnoses from a previous visit

The most important tab is the third one (Fig. 1), in which findings of the anterior and posterior segments of the eye, the intraocular pressure, and a treatment plan are entered. To capture the diagnoses, a table was placed in the lower right corner (blue box, Fig. 1). Three buttons for coding, which are described in the following sections, are placed next to the table (red box, Fig. 1).

### Diagnoses coding

Legal instructions require at least one treatment diagnosis per outpatient case to be documented. A case is defined as all the visits of one single outpatient during one quarter of a year. Every case carries a unique case number, and all visits during that quarter of the year are documented by using this case number. Diagnoses are documented by using the ICD-10 GM system [8].

Before the EHR was introduced, clinical findings were hand-written on lined paper together with a treatment plan in a separate column. A web-based tool developed by the hospital’s IT department was available on the hospital’s intranet in order to look up ICD-10 diagnoses by entering keywords. The diagnoses were hand-written on the first page of the patient record by an ophthalmologist and were then entered manually into the HIS by the administrative department.

Since the introduction of the EHR, the entry of a diagnosis in the PMD is mandatory for the treating physician. This task can be achieved either by using a hit list (Additional file 3: Figure S3) of the 50 most frequent diagnoses (3^rd^ button to be clicked on in red box in Fig. 1) or by means of a digital diagnoses search engine (ID DIACOS, ID GmbH, Berlin, Germany). This additional program, which was introduced alongside the implementation of the EHR, can be launched by clicking on the 2^nd^ button (red box in Fig. 1) in the findings and diagnoses tab of the GUI. If the patient has been to the hospital earlier, a previous diagnosis (Additional file 4: Figure S4) can be transferred from the preceding case by clicking on the fourth button next to the diagnoses table (red box in Fig. 1).

All ICD-10 diagnoses from the emergency room and from scheduled outpatient consultations before (January 2013 until June 2013) and after (January 2014 and June 2014) the introduction of the EHR in our department were obtained from the EHR system. The data were exported for every single week during these periods. Registered parameters were the average number of diagnoses per case, the various diagnoses per week, and the diagnoses made at least 5 times within a week. These parameters were stratified by patients who presented in the emergency unit without appointment and in the scheduled outpatient clinic. Additionally, the number of cases over the complete period was extracted and analyzed weekly. The weekly data of both periods were analyzed by using the Mann–Whitney–Wilcoxon test. A p-value of <0.05 was considered to be statistically significant. Diagnoses for each anatomical structure category of ICD-10-GM and for trauma conditions (see Legend of Table 1) were counted before and after the EHR introduction and plotted in a diagram.

## Results

### Number of cases

In the first period before the introduction of the EHR, 17,493 outpatient cases were recorded. After the introduction of an EHR in the second period, the number of outpatient cases was 18,538, revealing an increase of 5.97 %. Table 2 displays the detailed number of cases per week and unit (emergency unit and scheduled outpatient clinics). Significantly more cases per week were recorded in the emergency unit after the introduction of an EHR (*p* = 0.001). The absolute number of cases in our specialty outpatient clinics during both the periods was constant.

**Table 2:** Number of cases per week period 1 (P1) compared with period 2 (P2)

	Minimum		Maximum		Average		Median		SD		p
Cases per week:											
Period	P1	P2	P1	P2	P1	P2	P1	P2	P1	P2	
Emergency unit	266	308	385	446	323.9	359.1	318	362	31.72	37.19	0.001
Scheduled outpatients	237	206	534	527	355.12	360.24	331	345	46.79	45.31	0.58

### Variety of diagnoses

To investigate the spread and variety of diagnoses before and after the introduction of an EHR, the number of different diagnoses per week coded at least once for every patient was evaluated. In the first period, on average, 156.2 +/- 30.2 SD different diagnoses were made per week, and in the second period, an increase to 186.8 +/- 15.3 SD (*p* < 0.001) was observed.

To explore the variety of diagnoses further, we analyzed those diagnoses that were used at least 5 times a week. We found that in the paper-based documentation period, and in the digital documentation period, 34.3 +/- 9.0 SD and 47.5 +/- 6.0 SD respectively diagnoses were used for coding at least 5 times a week (*p* < 0.001).

Table 1 displays the change of diagnoses for each ICD-10 category and period. Four categories (diabetic complications in the eye (E14), visual acuity and eye examination (Z01.0), contusion of the globe (S05.1), and corneal injury (S05.0)) were coded less often, whereas the 15 other ICD-10 categories were used more frequently.

### Diagnoses per case

Comparing the weekly number of diagnoses during the observation periods, significant changes of the number of diagnoses per case were seen in the emergency unit and in the scheduled outpatient clinic. The number of diagnoses rose from 1.15 +/− 0.48 SD to 1.22 +/− 0.55 SD (*p* < 0.001) in emergency unit patient cases, and from 1.1 +/− 0.46 SD to 1.47 +/− 0.85 SD (*p* < 0.001) in scheduled outpatient cases (Table 3).

**Table 3:** Diagnoses per case period 1 (P1) compared with period 2 (P2)

	Minimum		Maximum		Average		Median			SD	p
Average number of diagnoses per case:											
Period	P1	P2	P1	P2	P1	P2	P1	P2	P1	P2	
Emergency unit	0	0	8	8	1.15	1.22	1	1	0.48	0.55	<0.001
Scheduled cases	8	8	7	7	1.10	1.47	1	1	0.46	0.85	<0.001

## Discussion

The introduction of the EHR optimized for ophthalmology led to a significant increase in the variety and number of documented diagnoses in our outpatient cases during the observational period.

We observed a significant change in the variety of coded diagnoses by the number of different diagnoses used per week (156.2 (+/−30.2 SD) vs. 186.8 (+/−15.3 SD)). As an online catalog with a full text search was available now, this option was probably used more often, leading to a more diverse coding behavior of the staff. Before this, with the old web-based software, only a very limited number of keywords could be used. When paper charts were still used, there was no need for a doctor to be logged into a PC, although they were available at any exam room. Consequently, the web-based tool was not used. Doctors may have used memorized ICD-10 codes. Rarer diagnoses codes may not be remembered by heart.

Before the introduction of the digital recording system, the administrative staff entered the diagnoses manually by transferring the code into the HIS that was hand-written in a file by the physician. However, vague codes such as “Z01.0,” which is “visual acuity testing,” were regularly entered in order to have at least one code with which to close the case and to fulfill requirements for the reimbursement. Sometimes, a physician did not provide a diagnosis in the paper chart. Moreover, in this situation, a Z01.0 diagnosis was possibly entered by hospital clerks. Notably, the number of diagnoses does not influence the amount of reimbursement for outpatients in Germany. The introduction of the digital system caused a sharp drop in this “wildcard” diagnosis (Table 1).

As the number of diagnoses per outpatient case also went up, the previously rarely documented diagnoses such as Z96.1 (presence of an intraocular lens implant), were used more often. The second largest increase in all diagnoses was related to lenses (ICD-10: H25-H28). Either of the two conditions (including aphakia H27.0) exists in every human. This is probably because more diagnoses are coded for every patient, and the lens status of a patient is documented more often.

The largest increase of diagnoses categories was in coding for choroidal and retinal diagnoses. The Department for Ophthalmology of the University of Munich is a specialized center for these kinds of maladies. Another confounder might be the increase in the number of patients being treated by intravitreal injections because of diseases such as age-related macular degeneration (AMD). In addition, early onset forms of AMD occur in many older patients. Meticulous physicians might have also coded this diagnosis, even though no treatment is necessary for it yet.

Rarer diagnoses such as corneal diseases (ICD-10 H15-H22) were probably coded more often, as the new online search engine offers a better tool for finding an appropriate diagnosis than the previously used self-made tool. It also contains an automatic correction for typographical errors and offers the most suitable diagnosis in cases for which no unique ICD-10 code for the medical condition is available.

One key issue in ophthalmology is the present lack of accuracy of ICD-10 in many ophthalmological diseases. Considerations were made in the past to differentiate ICD-10 coding further, but this has not been implemented as yet [9]. For example, all forms of AMD, be it neovascular AMD (“wet”) or geographic AMD (“dry”), are coded as H35.3 in ICD-10, although treatment approaches and courses of diseases differ greatly [10]. For clinical studies, a more detailed classification is necessary. To achieve this, the World Health Organization (WHO) currently proposes an ICD-11 draft online in which professionals are asked to participate and give their input for a more precise and up-to-date classification of diseases [11]. At present, the ICD-11 draft includes its own subgroup for macular diseases (ICD-11: AG40).

Before the introduction of EHR, differences in diagnoses per case between scheduled patients and emergency patients were minimal (1.10 vs. 1.15). Once EHR was implemented, diagnoses of emergency unit patients increased to 1.20 diagnoses per case. In scheduled patients, the increase was even more intense: It went up from 1.08 to 1.47 diagnoses per case. Two explanations for this finding could be: (1) the complexity of scheduled patients in an academic center and (2) the consultants now being part of the coding process to close the case. An emergency care of patients is often more straightforward. Additionally, residents often treat only emergency patients, especially in less complex situations in which a single diagnosis is usually sufficient.

A major concern raised by critics about EHR is the increase in the time needed for documentation and the consecutive reduction of patient treatment time, resulting in reduced numbers of patients treated [12]. However, the introduction of the digital system did not result in a decay of treated patients in our department; on the contrary, the overall number of treated patients increased by 5.97 %. The general higher case numbers can cause an increase in the total number of diagnoses per period but will not alter the number of codes per case. The medical workforce in our department was not altered during the study period. One explanation for the increased patient numbers could be that, in the second period, every consultation had to be documented digitally, which is only possible, if a valid visit in the HIS on that day occurs. Before the introduction of an EHR, consultations without valid cases in the HIS might have taken place.

At present, not all of the subspecialties covered in our department are integrated into the digital system [6].

As the EHR training of staff took place, it might have led to a bias in the results, although neither any specific education on diagnoses coding was provided for any staff group nor were the advantages of coding promoted. It might be possible that training caused more attention to proper coding. Another limitation of this study due to labor privacy laws in Germany was that we were not able to investigate the individual physician's contributions to the coding changes, which in theory could explain some of the changes in coding found in this manuscript.

## Conclusions

In this study, we have demonstrated that a significant shift occurs in diagnoses after the introduction of an EHR. The number of diagnoses per case rise significantly in emergency department patients. In addition to the change in numbers, the diversity of diagnoses alters. For research purposes, it is advantageous to have as many precise diagnoses recorded as possible. It will greatly facilitate queries in an EHR for patients. In the future, many possibilities may arise when using EHR data for research [13–15].

## Abbreviations

ABAP, advanced business application programming; AMD, age-related macular degeneration; COBOL, common business oriented language; EHR, electronic health record; ERP, enterprise resource planning; ETDRS, early treatment of diabetic retinopathy study; GUI, graphical user interface; HIS, hospital information system; ICD-10, international classification of diseases version 10; ICD-10-CM, international classification of diseases version 10 clinical modification; ICD-10-GM, international classification of diseases version 10 German modification; ICD-11, international classification of diseases version 11; LMU, Ludwig-Maximilian University Munich; PMD, parametric medical documents; SD, standard deviation; WHO, World Health Organization

## Additional files

Additional file 1: Figure S1. Current complaint and past medical history tab. (PNG 30 kb) Additional file 2: Figure S2. Visual acuity tab. (PNG 86 kb) Additional file 3: Figure S3. Diagnoses hit list, containing the 50 most often used diagnoses. (PNG 30 kb) Additional file 4: Figure S4. Previous diagnoses of the patient. They can be selected and transferred to the current case. (PNG 37 kb)
