# Supplementary figures and images for: The influence of a specific ophthalmological electronic health record on ICD-10 coding
Source: BMC Med Inform Decis Mak. 2016 Jul 26;16:100. doi: 10.1186/s12911-016-0340-1 (PMC4962360; doi:10.1186/s12911-016-0340-1)

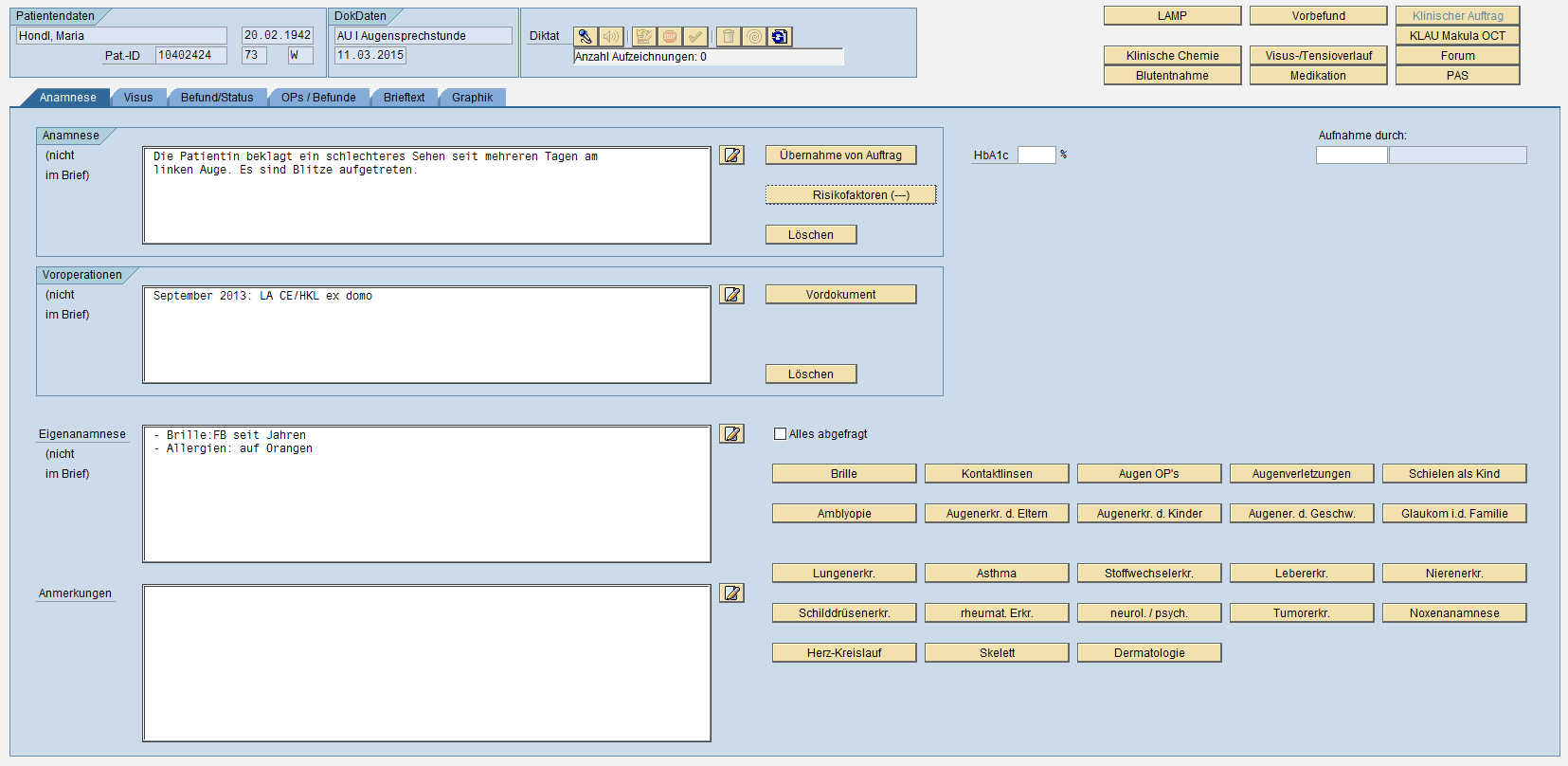

Supplement: Additional file 1: Figure S1. — Current complaint and past medical history tab. (PNG 30 kb) [file 12911_2016_340_MOESM1_ESM.png]

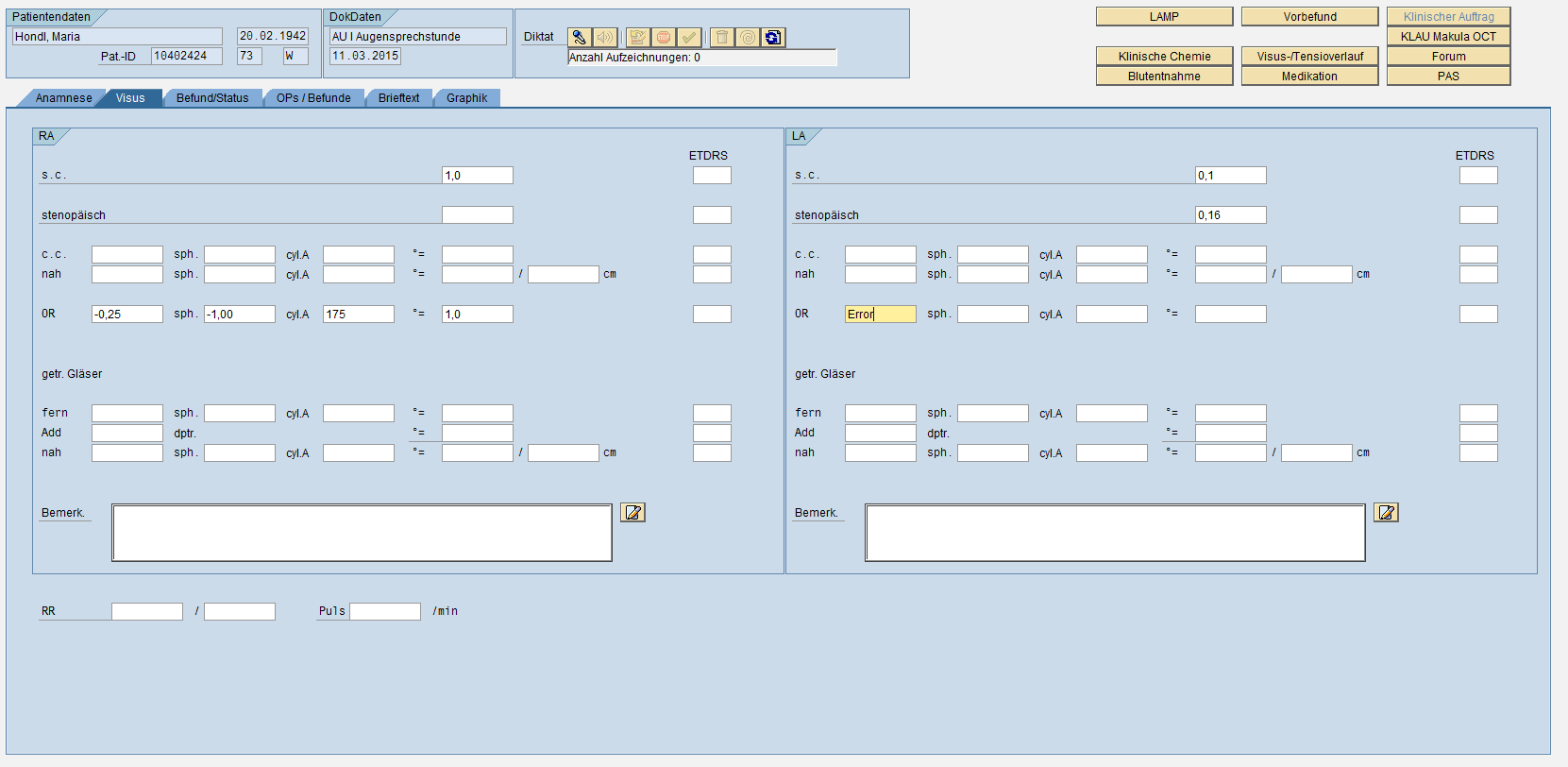

Supplement: Additional file 2: Figure S2. — Visual acuity tab. (PNG 86 kb) [file 12911_2016_340_MOESM2_ESM.png]

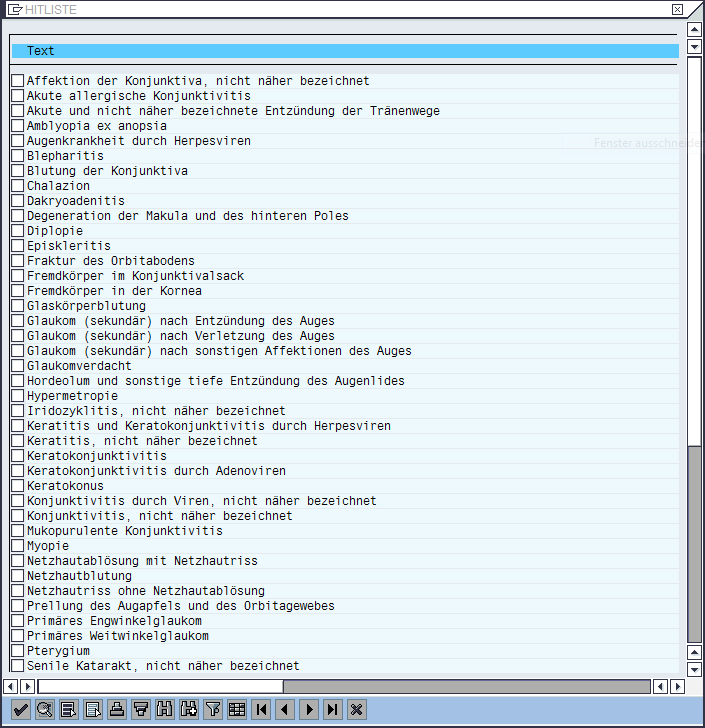

Supplement: Additional file 3: Figure S3. — Diagnoses hit list, containing the 50 most often used diagnoses. (PNG 30 kb) [file 12911_2016_340_MOESM3_ESM.png]

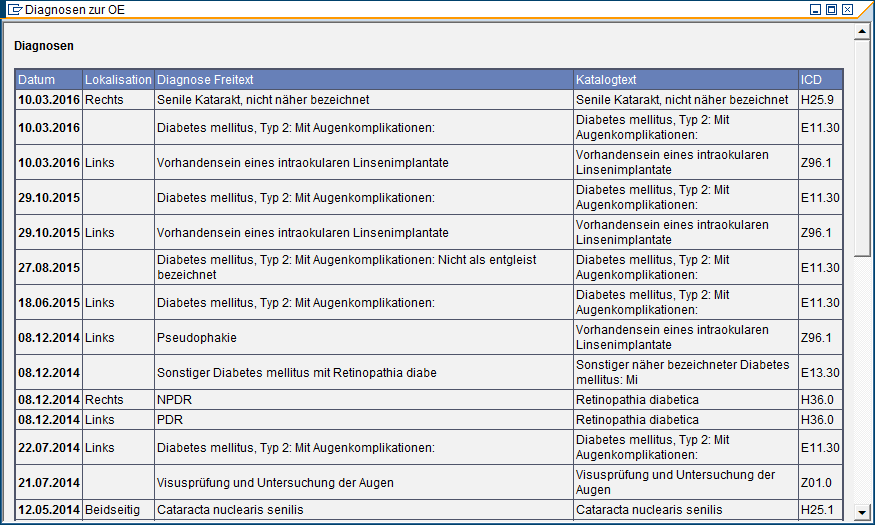

Supplement: Additional file 4: Figure S4. — Previous diagnoses of the patient. They can be selected and transferred to the current case. (PNG 37 kb) [file 12911_2016_340_MOESM4_ESM.png]
